# Transcriptome and selected metabolite analyses reveal points of sugar metabolism in the developing leaves of kiwifruit

**DOI:** 10.3389/fpls.2025.1618801

**Published:** 2025-07-11

**Authors:** Yacheng Huang, Kaixiu Jiang, Huanyan Liu, Shuyi Song, Yanmei Zhao, Bin He, Jixian Lan, Linya Liu

**Affiliations:** ^1^ School of Biological Science and Technology, Liupanshui Normal University, Liupanshui, China; ^2^ College of Bioscience and Biotechnology, Shenyang Agricultural University, Shenyang, China

**Keywords:** kiwifruit, leaf development, sucrose metabolism, sugar content, enzyme activity, comparative transcriptome

## Abstract

In fruit crops, sugars are essential metabolites, that are produced in leaves and subsequently transported to fruits. However, the sugar levels, gene expression patterns, and sucrose-metabolizing enzyme activities in the leaves of kiwifruit remain poorly understood. In this study, the Illumina NovaSeq 6000 platform was utilized to sequence the kiwifruit leaf transcriptome at 4 developmental stages (A-D), which yielded 109,832 unigenes (mean length, 1135 bp). In addition, the sugar-related genes were compared for their expression profiles and their associations with sugar accumulation and enzyme activities in kiwifruit leaves during growth. The fructose content increased from stages A to C and declined in stage D (mature leaf stage), but the glucose, sucrose, and starch contents increased continuously throughout the leaf development period. The gene expression patterns and sucrose-metabolizing enzyme activities in kiwifruit leaf samples exhibited variations from those of other plant species. Sucrose synthase was revealed as the primary enzyme for sucrose breakdown during early leaf development (stages A and B), whereas cytoplasmic invertase and cell wall invertase exhibited activities comparable to those of vacuolar invertase in the later stages of leaf development (stages C and D), which is consistent with the transcriptional changes noted in most of their encoding genes. On the other hand, sucrose synthase, operating in the synthetic direction, exhibited greater activity than sucrose phosphate synthase across all leaf developmental stages. Overall, these results shed more light on the molecular mechanisms associated with sugar metabolism in kiwifruit leaves.

## 1 Introduction

Leaf development is a meticulously orchestrated process, involving a cascade of morphological, physiological, and biochemical transformations (Ribeiro et al., 2021; Lv et al., 2023). In the early stages of leaf development, rapid cell division and expansion necessitate a high influx of hexoses, such as glucose and fructose, which are employed directly for energy production and biosynthetic processes (Koch, 2004; Rolland et al., 2006). As leaves transition to maturity, sucrose becomes the predominant form of translocated sugar, acting as a primary carbon carrier to non-photosynthetic tissues and a regulator of metabolic function in the plant (Lambers et al., 2019; Ribeiro et al., 2021). Consequently, healthy leaf generation and maturation play key roles in plant development and defense mechanisms as well as in achieving optimal agricultural productivity. Each stage of leaf development has unique metabolic requirements and sugar utilization patterns, which underscores the intricate coordination of sugar metabolism, driven by specific enzyme activities and associated gene expression levels (Ruan, 2014).

Sucrose synthase (SuSy), invertase (IVN), sucrose phosphate phosphatase (SPP), sucrose phosphate synthase (SPS), hexokinase (HK), and fructokinase (FK) are enzymes that play indispensable roles in the modulation of sucrose synthesis, degradation, and utilization during leaf development (Park et al., 2012). Over the past 30 years, great advancements have been made in elucidating the changes in and the roles of sucrose-metabolizing enzymes throughout the process of leaf development (Turgeon, 1989; Foyer and Paul, 2001; Ruan, 2014). Sucrose synthase (SuSy) and invertase (INV) are the key enzymes involved in sucrose breakdown, resulting in the generation of hexoses, which are essential for leaf growth. SuSy facilitates the reversible conversion of UDP and sucrose to UDP-glucose and fructose, whereas INV irreversibly catalyzes sucrose hydrolysis to fructose and glucose. INV can be categorized into three isoforms according to their respective subcellular location and optimal pH: cell wall invertase (CWI), vacuolar invertase (VIN), and cytoplasmic invertase (NIN) (Tymowska-Lalanne and Kreis, 1998). In numerous plant species, VIN reportedly exerts an important effect on the cleavage of sucrose in growing leaves, whereas NIN and SuSy exhibit comparable cleaving effects on *Hevea* (Zhu et al., 2018). Sucrose can be synthesized not only by SuSy, which catalyzes the UDP-glucose and fructose reactions, but also by UDP-glucose and fructose-6-phosphate through SPP and SPS, respectively. In general, when sucrose synthesis is prioritized, SuSy expression is often heightened during the phases of rapid cell expansion, whereas SPS is crucial in later leaf development stages when sucrose accumulation must be exported to other plant parts, as reported for rice leaves and sugar beet leaves (Pavlinova et al., 2002; Hashida et al., 2016). However, at various leaf development stages of *Hevea*, SuSy activity in the synthesis direction (SSS) is reportedly similar to or superior to that of SPS (Zhu et al., 2018). In addition, hexokinase (HK), a glucose-fructose-phosphorylating enzyme, can convert glucose into D-glucose-6-phosphate, which serves to catalyze the initial glucose metabolism step (Saltman, 1953), whereas fructokinase (FK) catalyzes fructose phosphorylation into fructose-6-phosphate, which is a critical step in fructose metabolism (Copeland et al., 1984). Both of these enzymes play crucial roles in regulating sugar utilization and energy homeostasis in plant leaves under both physiological conditions and stress conditions (Granot et al., 2013). In recent studies, transgenic and gene-silencing approaches have been employed to explore the roles of sucrose-metabolizing enzymes in leaf development. For example, overexpression of the potato SuSy gene in transgenic cotton resulted in faster expansion of young leaves, along with significantly increased fructose content and slightly reduction in sucrose concentration (Xu et al., 2012). Similarly, transgenic *Arabidopsis* plants with altered SuSy expression revealed the essential role of this enzyme in regulating carbon partitioning and maintaining cell wall integrity, highlighting the contribution of SuSy to leaf growth and development (Yao et al., 2020). Furthermore, tomato plants with silenced sucrose phosphate synthase (SPS) genes were reported to have reduced sucrose accumulation and altered carbohydrate partitioning, which underscores the importance of SPS in sucrose synthesis and its influence on leaf development (Galtier et al., 1993). The activities of these enzymes, as evidenced and collectively emphasized by the above findings, are finely tuned and exhibit significant variations across different developmental stages, mirroring the evolving metabolic requirements of the leaf (Koch, 2004). Therefore, it is worthwhile to study how the relative importance of sucrose-metabolizing enzymes varies across different plant organs, species, and developmental stages.

Kiwifruit (*Actinidia chinensis*) is an important fruit crop worldwide that is valued for its remarkable economic, nutritional, and health benefits (Ferguson, 2014). In recent years, Hongyang kiwifruit, an *A. chinensis* var. *chinensis* variety (Wang et al., 2003), has gained popularity among consumers, farmers, and breeders because of its distinctive characteristics, resulting in a relatively high price on the market. In most fruit crops, sucrose is the major carbon transportation type and is produced and transported from the leaf (source organ) to the fruiting body (sink organ) (Ruan, 2014; Li et al., 2018), thereby affecting fruit growth and quality. However, few studies have investigated the physiological and molecular modulation of sucrose metabolism in kiwifruit leaves. The present work focused on exploring the dynamics of sugar metabolism by determining and analyzing alterations in sugar levels, metabolic enzyme activities, and the transcriptome in four developmental stages of kiwifruit leaves. Integrating physiological and molecular data, this study aimed to elucidate the regulatory mechanisms underlying sugar metabolism and their role in leaf development. The results are expected to assist in comprehensively understanding the association of sucrose-metabolizing enzyme activities with transcript levels in the four developmental stages of kiwifruit leaves, offering valuable perspectives for kiwifruit improvement.

## 2 Materials and methods

### 2.1 Plant materials

**Figure 1:**
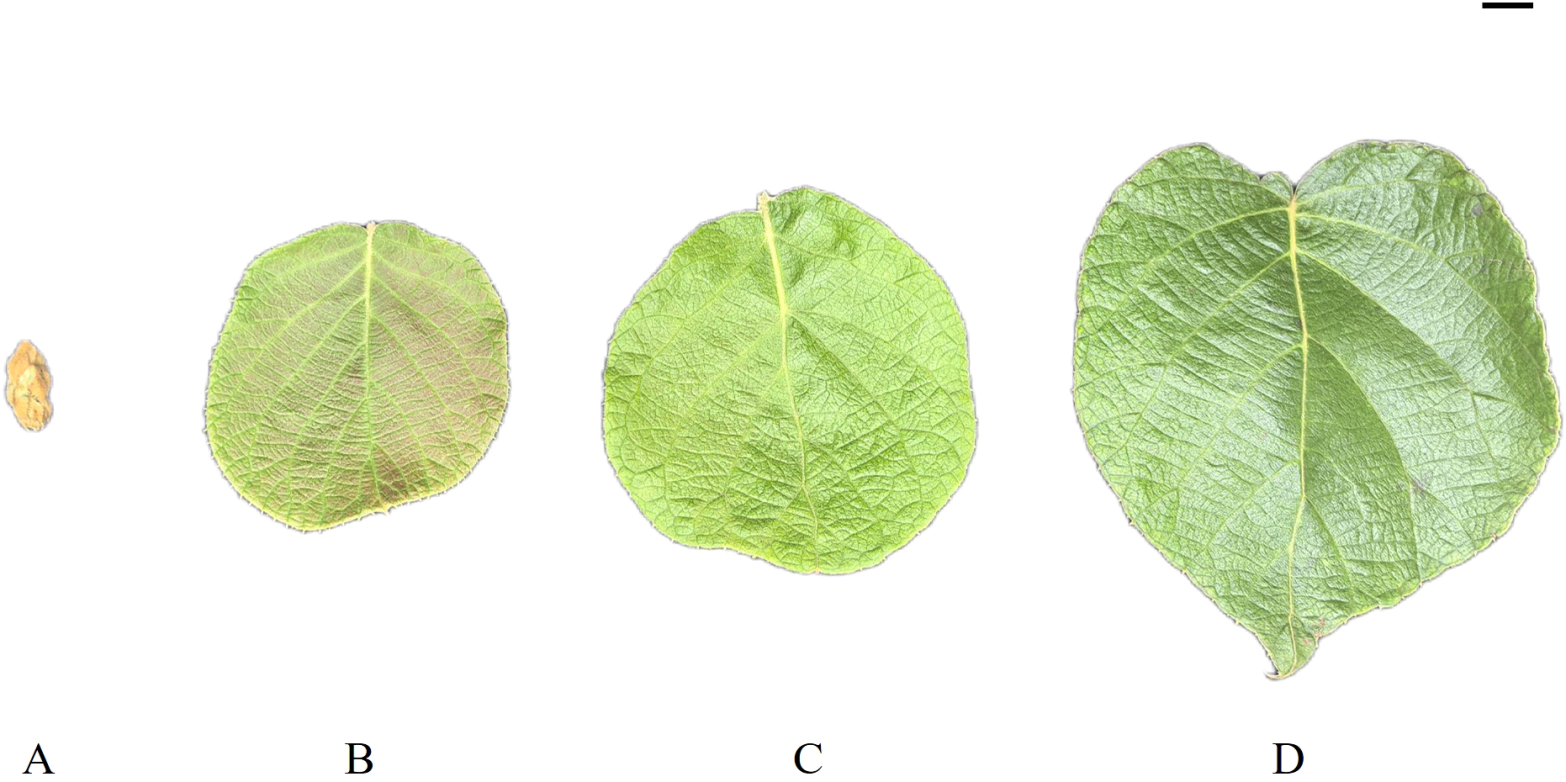
Leaf development stages. Four developmental stages of kiwifruit leaf. **(A)** bud; **(B)** bronze; **(C)** pale-green; **(D)** mature.

The 8-year-old kiwifruit cultivar Hongyang was planted in an experimental field in Liupanshui City, Guizhou Province, China, in 2023. The coordinates of the specific location were 104.55E, 26.25 N. Leaves from four developmental stages, i.e., bud (A), bronze (B), pale-green (C), and mature (D) (**Figure 1**), were collected from the fruiting branch of kiwifruit trees at approximately 38 days after full bloom. The leaf development from bud (A) to mature (D) in Hongyang kiwifruit lasts a period of ~35d, with 10~15d being bronze and 8~10d being pale-green. Three biological replicates were obtained for each sample, and 5 leaves were randomly collected from trees in the same plot of the orchard. All the collected leaves were immediately frozen in liquid nitrogen and stored at –80°C until used.

### 2.2 Carbohydrate analysis

Leaf samples were subjected to 30 min of desiccation at 105°C and later at 80°C to obtain a constant weight. Soluble sugar extraction was completed as described by Ma et al. (2014) with certain specific modifications. All samples (in known quantities) were ground into powder, and 5 mL of the extraction solvent (80% ethanol solution) was added to this powder, followed by 30 min of incubation at 80°C with shaking every 5 min. The supernatants were obtained through centrifugation (4°C, 20,000 ×g, 10 min), and 5 mL of the extraction solvent was added again to further extract the pellet. Later, the supernatants collected from both centrifugation steps were subjected to vacuum drying at 60°C, after which the dried samples were dissolved in deionized water and subsequently stored at –20°C before analysis. Sucrose, D-glucose, and D-fructose assay kits (Megazyme) were subsequently used in accordance with specific protocols to quantify the sugar content. A Total Starch Assay Kit (Megazyme, Bray, Ireland) was used to extract starch and analyze its content in kiwifruit leaf samples.

### 2.3 Assays to determine enzyme activities

Fresh leaves preserved at –80°C were ground with ice. Later, the samples were accurately weighed (0.1 g) and used for determining the enzyme activities at the tissue mass-to-extraction solution ratio of 1:5–10 (v/v). SuSy, SPS, NIN, CWI, and VIN activities were detected with SSS (SSII-1-Y), SSC (SSI-1-Y), SPS (SPS-1-Y), NIN (NI-1-Y), CWI (BAI-1-Y), and VIN (SAI-1-Y) assay kits provided by Suzhou Grace Biotechnology Co. Ltd. (Suzhou, China). Each experimental procedure was conducted three times.

### 2.4 Total RNA extraction, cDNA library construction, and sequencing

Total RNA was extracted via a modified CTAB method (Liu et al., 2020) and then treated with RNase-free DNase I (Ferments Inc.). The RNA integrity, concentration, and purity were monitored using an Agilent 2100 Bioanalyzer and analyzed using 1.5% agarose gel electrophoresis. Twelve high-quality RNA samples were obtained from Novogene Biotech. Co. Ltd. (Beijing, China) for constructing the cDNA libraries using the NEBNext^®^ Ultra™ RNA Library Prep Kit for Illumina^®^ (NEB, USA) in accordance with specific instructions followed by sequencing using the Illumina NovaSeq 6000 platform with paired-end sequencing.

### 2.5 The *de novo* transcriptome assembly and unigene annotation

After cDNA library sequencing, CASAVA v1.8. software was used for transforming the obtained raw image data into sequence information and then estimating the sequencing read quality with FastQC. Adapter and low-quality sequences, as well as ambiguous reads, were removed to acquire high-quality clean reads (>90% of Q≥20 bases). Thereafter, the clean reads were assembled using the Trinity program, and non-redundant unigenes (≥300 bp in length) were obtained from 12 clean datasets (Grabherr et al., 2011). After assembly, a BLAST search against various nucleotide and protein databases, such as the Nr, Nt, Pfam, KO, KOG, Swiss-Prot, and Kiwifruit Genome databases (http://kiwifruitgenome.org/) (Yue et al., 2020), was performed to annotate the unigenes, and an e value < 1e^–5^ was set as the significance level. On the basis of the Nr and Pfam annotations, a Gene Ontology (GO) analysis of the unigenes was carried out using Blast2GO software (Conesa et al., 2005), and pathway enrichment was performed using the Kyoto Encyclopedia of Genes and Genomes (KEGG) database.

### 2.6 DEG identification and enrichment

In order to identify the DEGs (P value ≤ 0.05, |log_2_fold-change| ≥ 1, FDR < 0.01) across the four samples analyzed in this study, the high-quality clean reads were mapped to the unigenes using Bowite2 with default parameters (Robinson et al., 2010). Subsequently, the RSEM software was utilized to compute the reads per kilobase per million reads (RPKM) value for each unigene in the four tissues (Li and Dewey, 2011) to determine the gene transcript abundance according to Mortazavi et al.’s method (2008). The upregulated and downregulated DEGs were subjected to GO and KEGG analyses using topGO (http://www.bioconductor.org/) and KOBAS (Mao et al., 2005) (KEGG Orthology-Based Annotation System), respectively, with an FDR < 0.05 as the criterion.

### 2.7 Validation of the identified DEGs using qRT-PCR

Twenty DEGs were selected on the basis of their different expression levels and analyzed using qRT-PCR to confirm the DEG-based analysis results. After the total sample of RNA was extracted, the first-strand cDNA was synthesized using a reverse transcription kit (Takara, Tokyo, Japan), following specific protocols. The DEG primers used were designed with Primer Premier 5.0, and a melting curve analysis was performed. Finally, the amplified fragments were sequenced to verify their specificity. TB Green^®^ *Premix Ex Taq* ^™^ II (Tli RNaseH Plus) (Takara, Tokyo, Japan) and a Bio-Rad CFX connect real-time PCR detection system were used, and the method described previously (Liu et al., 2024) was used to perform qRT-PCR. The 2^–ΔΔCt^ approach was employed for determining the gene expression level (Trick et al., 2021), with *GAPDH* and *18S* used as the internal control genes. **Supplementary Table 1** displays all the gene-specific primers used in this study.

### 2.8 Statistical analysis

All the statistical analyses were conducted using one-way ANOVA in GraphPad Prism 8.0, and the values indicated with different letters represent significant differences (p < 0.05). Correlation coefficients were determined between the activity of sucrose-metabolizing enzymes and gene expression during the course of kiwifruit leaf development. Correlation was accepted as significant at P < 0.05 or 0.01.

## 3 Results

### 3.1 Alterations in sugar composition during leaf development in kiwifruit

**Figure 2:**
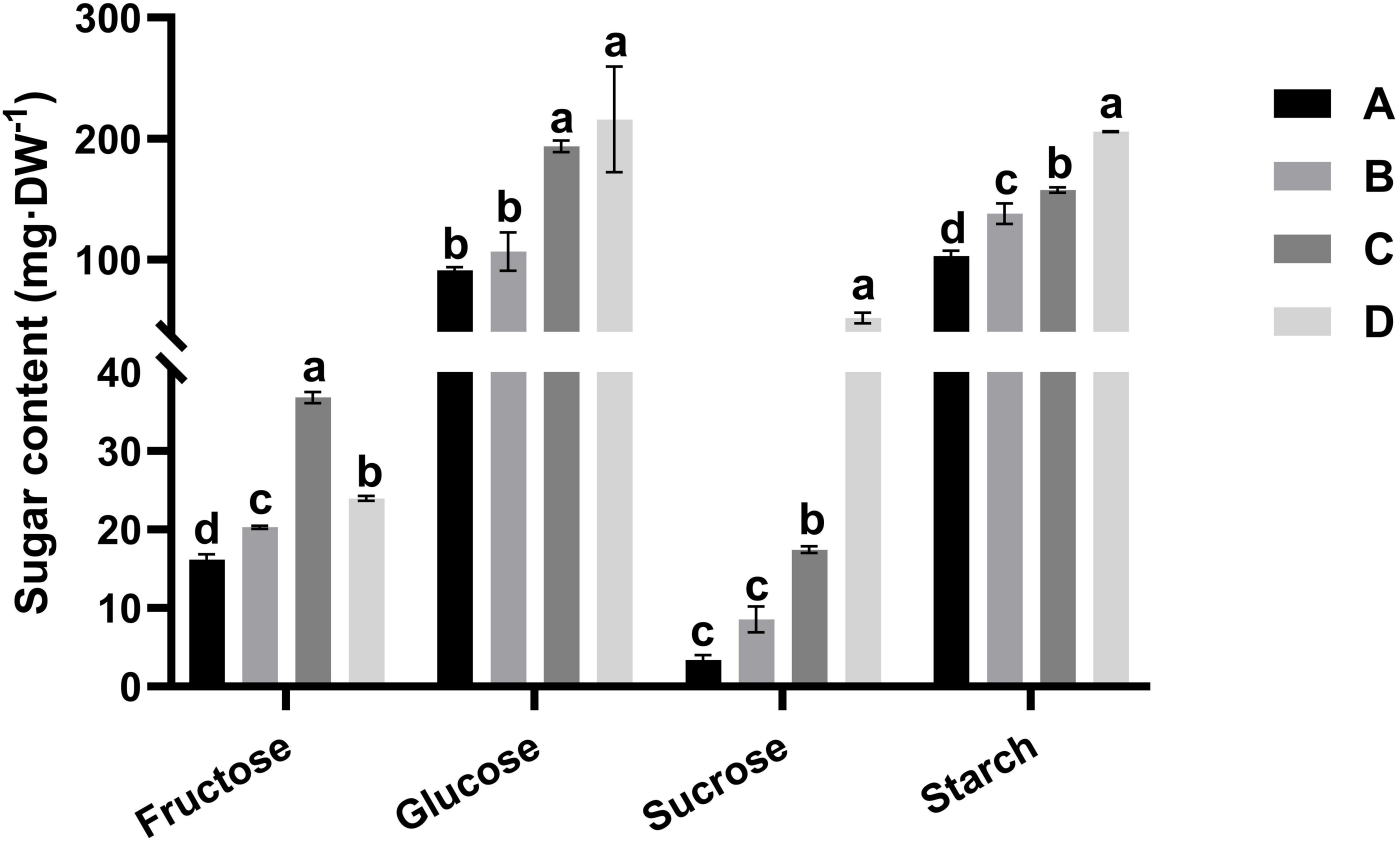
Alterations in the fructose, glucose, sucrose, and starch contents in the four leaf development stages of kiwifruit **(A–D)**. The results presented are the means ± SEs from three replicates. Different letters above the bars indicate a difference that is significant (P < 0.05) across different leaf development stages. Four developmental stages of kiwifruit leaf. **(A)** bud; **(B)** bronze; **(C)** pale-green; **(D)** mature.

To determine the sugar composition of the kiwifruit leaf samples, the contents of sucrose, glucose, fructose, and starch were determined for each leaf developmental stage. **Figure 2** displays the alterations in the fructose, glucose, sucrose, and starch contents during leaf development. Starch accumulation was detected throughout all stages of leaf development, with starch content in stage D reaching approximately twice the level recorded in stage A. Notably, the starch content was much greater than the sucrose content at all leaf stages. In stage D (maturation stage), the ratio of starch to sucrose was about 4.0, consistent with values reported in the leaves of many plant species. The concentration of fructose increased progressively throughout the development process, doubling in stage C compared to the level in stage A and later declining sharply in the maturation stage. The glucose and sucrose contents did not change during stages A–B but markedly increased in stage C, prior to reaching the peak value in stage D. Specifically, despite a 15-fold increase in sucrose from stage A to stage D, glucose consistently remained at the highest concentration among the soluble sugars during all stages of leaf development.

### 3.2 Activities of key enzymes involved in sugar metabolism during leaf development

**Figure 3:**
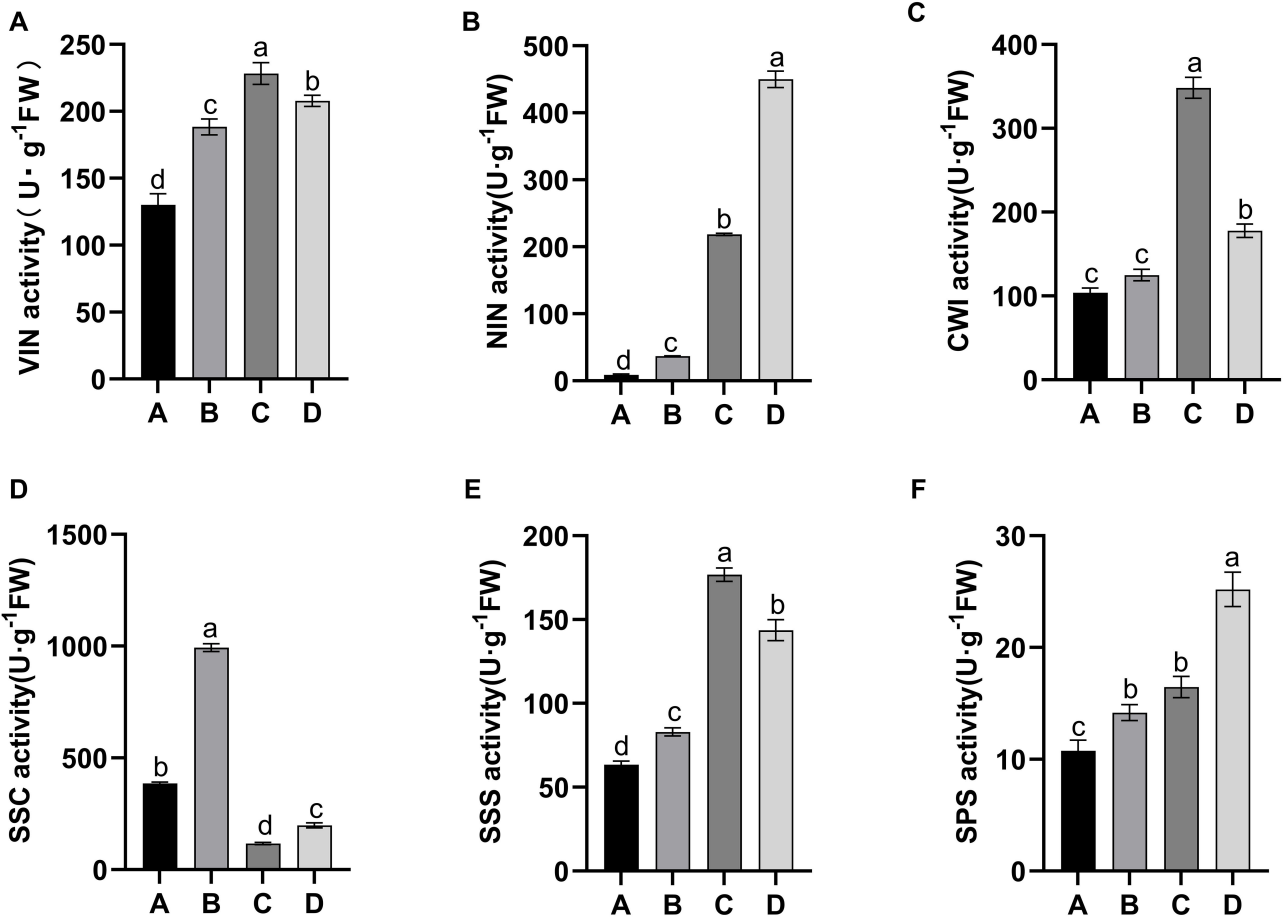
Alterations in the activities of sucrose-metabolizing enzymes in four leaf development stages of kiwifruit. **(A)** vacuolar invertase (VIN); **(B)** alkaline/neutral invertase (NIN); **(C)** cell wall invertase (CWI); **(D)** sucrose synthase in the cleavage direction (SSC); **(E)** sucrose synthase in the synthesis direction (SSS); **(F)** sucrose phosphate synthase (SPS). The results presented are the means ± SEs from three replicates. The different letters on the top of the bars indicate a significant difference (P < 0.05) across the different leaf development stages. Four developmental stages of kiwifruit leaf. **(A)** bud; **(B)** bronze; **(C)** pale-green; **(D)** mature.

Sucrose-cleaving enzymes, including VIN, NIN, CWI, and SuSy, in the cleavage direction (SSC)exhibited similar activities in the growing leaf samples (stages A–D). The activities of these four enzymes, for example, changed significantly (**Figure 3**). The alterations in VIN, NIN, and CWI activities were similar and slowly increased from stage A to stage C (**Figures 3A–C**). At stage D, NIN activity increased further, but VIN and CWI activities decreased (**Figures 3A–C**). In addition, the activity of SuSy differed from that of invertase, with significantly higher activity noted in stages A–B than in stages C–D (**Figure 3D**). Further comparison of the activities of these four enzymes during the same developmental stage revealed that NIN presented the highest activity at stage D, and SSC presented the highest activity at stages A and B (**Figures 3B–D**). In contrast, VIN, NIN, and CWI displayed elevated activities at stage C (**Figures 3A–C**). These findings suggested that sucrose decomposition at different stages of leaf development in kiwifruit may be driven by distinct types of enzymes. Sucrose-synthesizing enzymes, namely, SPS and SuSy, in SSS, exhibited increased activity during leaf development, but SuSy activity markedly increased relative to that in SPS (**Figures 3E, F**).

### 3.3 Illumina sequencing, assembly, and gene annotation

In order to obtain the transcriptome profiles of kiwifruit leaves, cDNA libraries were constructed for the four leaf development stages, followed by sequencing using the Illumina NovaSeq 6000 platform. After eliminating the adaptor sequences and low-quality reads, 725,193,794 clean pair-end reads were obtained from the 12 cDNA libraries. A total of 109,832 unigenes (N_50_, 1839 bp; mean length, 1135 bp; minimum length, 301; maximum length, 16,929) were assembled using the Trinity software. Among these unigenes, 39,866 unigenes were within the length range of 200–400 bp, 32,396 were within 500–1000 bp, 20,011 were within 1000–2000 bp, and 17,559 unigenes exceeded 2000 bp in length (**Table 1**). The unigene length distribution results revealed the inverse proportion of quantity to length.

**Table 1:** Sample transcriptome data assembly.

Assembly	Number
Number of clean reads (Bud)	208,721,082
Number of clean reads (Bronze)	166,963,738
Number of clean reads (Pale-green)	167,559,020
Number of clean reads (Mature)	181,949,954
Number of Unigenes	109,832
Unigenes (300–500 bp)	39,866
Unigenes (500–1000 bp)	32,396
Unigenes (1000–2000 bp)	20,011
Unigenes (>2000 bp)	17,559
N50 length (bp)	1,839
Mean unigene length(bp)	1,135

**Figure 4:**
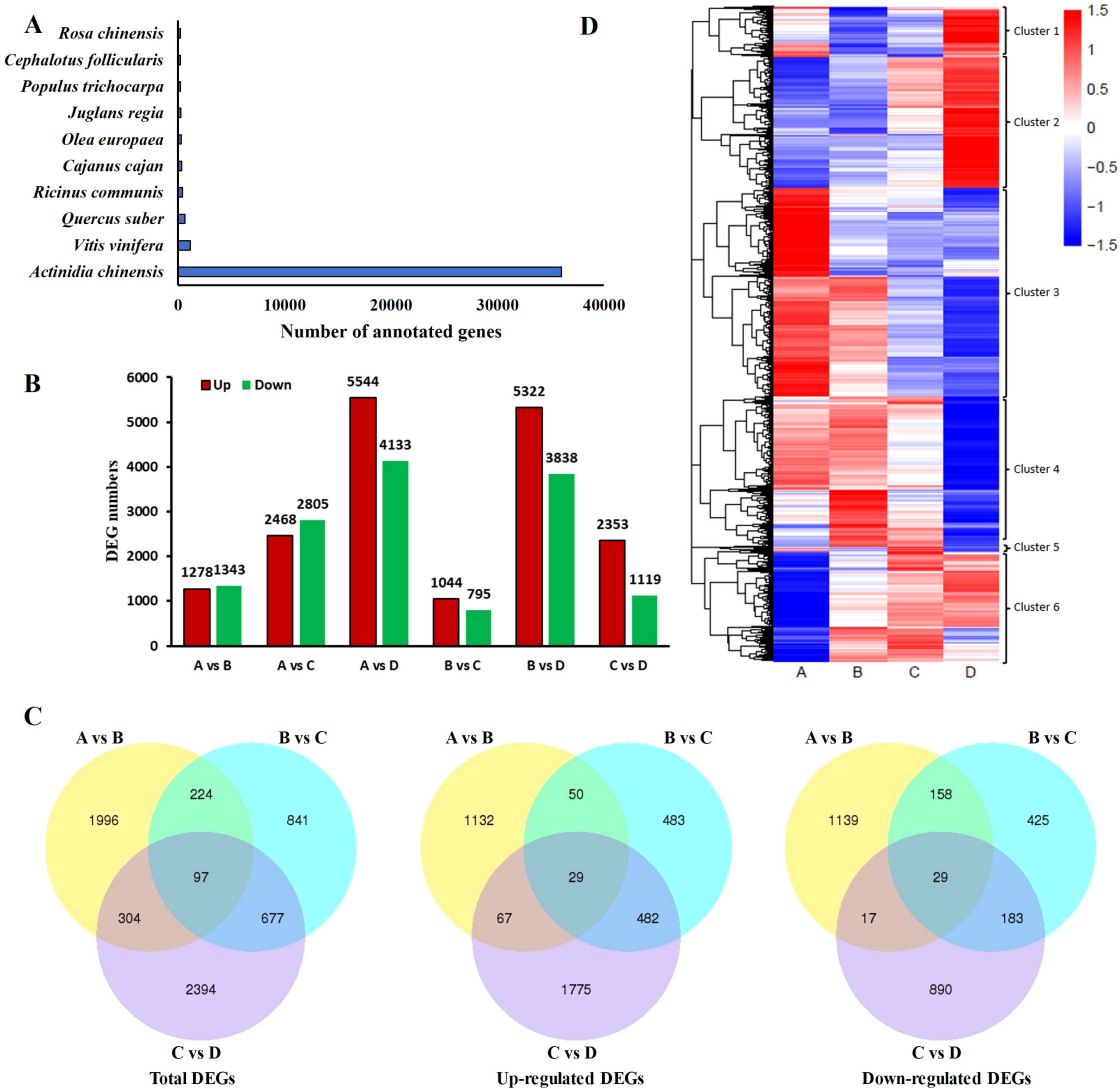
The differentially expressed genes (DEGs) were identified through transcriptome sequencing using samples from four leaf development stages in kiwifruit. **(A)** Number of top hits among species from the BLASTX results of searches against the Nr database. **(B)** Summary of DEGs in all combinations of leaf development stage comparisons. **(C)** Venn diagram comparing the DEGs between A and B, B and C, and C and D (|log_2_fold-change| ≥ 1 and false discovery rate (FDR) ≤ 0.01). **(D)** Heatmap hierarchical clustering for the identified DEGs. Four developmental stages of kiwifruit leaf. **(A)** bud; **(B)** bronze; **(C)** pale-green; **(D)** mature.

In order to annotate the unigenes identified in the kiwifruit leaf samples, a BLAST search against public databases such as NR, Nt, Swiss-Prot, GO, KEGG, PFAM, and EuKaryotic Orthologous Groups (KOGs) was conducted. Thereafter, the functional data of the proteins obtained were assigned to every unigene according to the greatest score (optimal hit). There were 44372 (40.4%), 35124 (31.98%), 32103 (29.23%), 34036 (30.99%), 17353 (15.8%), 34036 (30.99%), and 8940 (8.14%) unigenes annotated using NR, NT, Swiss-Prot, GO, KEGG, PFAM and KOG, respectively. Moreover, among the 69,741 unigenes, 58,379 (53.15%) genes were successfully annotated using one or more hits, whereas 4,993 (4.54%) were matched to these seven databases (**Table 2**). In addition, among the 44 unigenes, 373 unigenes of kiwifruit annotated in the Nr database had homologs in numerous plant species. *Actinidia chinensis*, *Vitis vinifera*, and *Quercus suber* were the top three plant species, with annotations of 35,997 (81.12%), 1,156 (2.61%), and 636 (1.43%) unigenes, respectively (**Figure 4A**). In the GO annotation, 34,038 unigenes were associated with three categories: biological process (85,650 hits), cellular component (54,002 hits), and molecular function (38,610 hits). In the biological process (BP) category, these genes were associated with 26 GO terms, with ‘cellular process’, ‘metabolic process’, and ‘single-organism process’ being the three significantly enriched subcategories. In the CC category, the genes matched 20 GO terms, and the largest subcategories were ‘cell’ and ‘cell part’. In the molecular function (MF) category, the two most abundant subcategories were ‘binding’ and ‘catalytic activity’, which contained 10 GO terms (**Supplementary Table 2**). The KEGG annotation system was utilized to determine the cell metabolic pathways and functions of gene products through a mapping of the assembled unigenes. A total of 17,357 unigenes were mapped to 131 KEGG pathways, with the ‘carbohydrate metabolism’ (1,400 unigenes), ‘translation’ (1,293 unigenes), and ‘folding, sorting and degradation’ (1,118 unigenes) pathways presenting the most unigenes (**Supplementary Table 3**).

**Table 2:** Annotation statistics of the kiwifruit leaf transcriptome.

Annotation	Mapped unigenes
Annotation in NR	44,373(40.40%)
Annotation in NT	35,130(31.98%)
Annotation in KEGG	17,357(15.80%)
Annotation in SwissProt	32,107(29.23%)
Annotation in PFAM	34,038(30.99%)
Annotation in GO	34,038(30.99%)
Annotation in KOG	8,951(8.14%)
Annotation in all Databases	4,993(4.54%)
Annotation in one or more Databases	58,379(53.15%)

### 3.4 GO and KEGG analyses of DEGs

**Figure 5:**
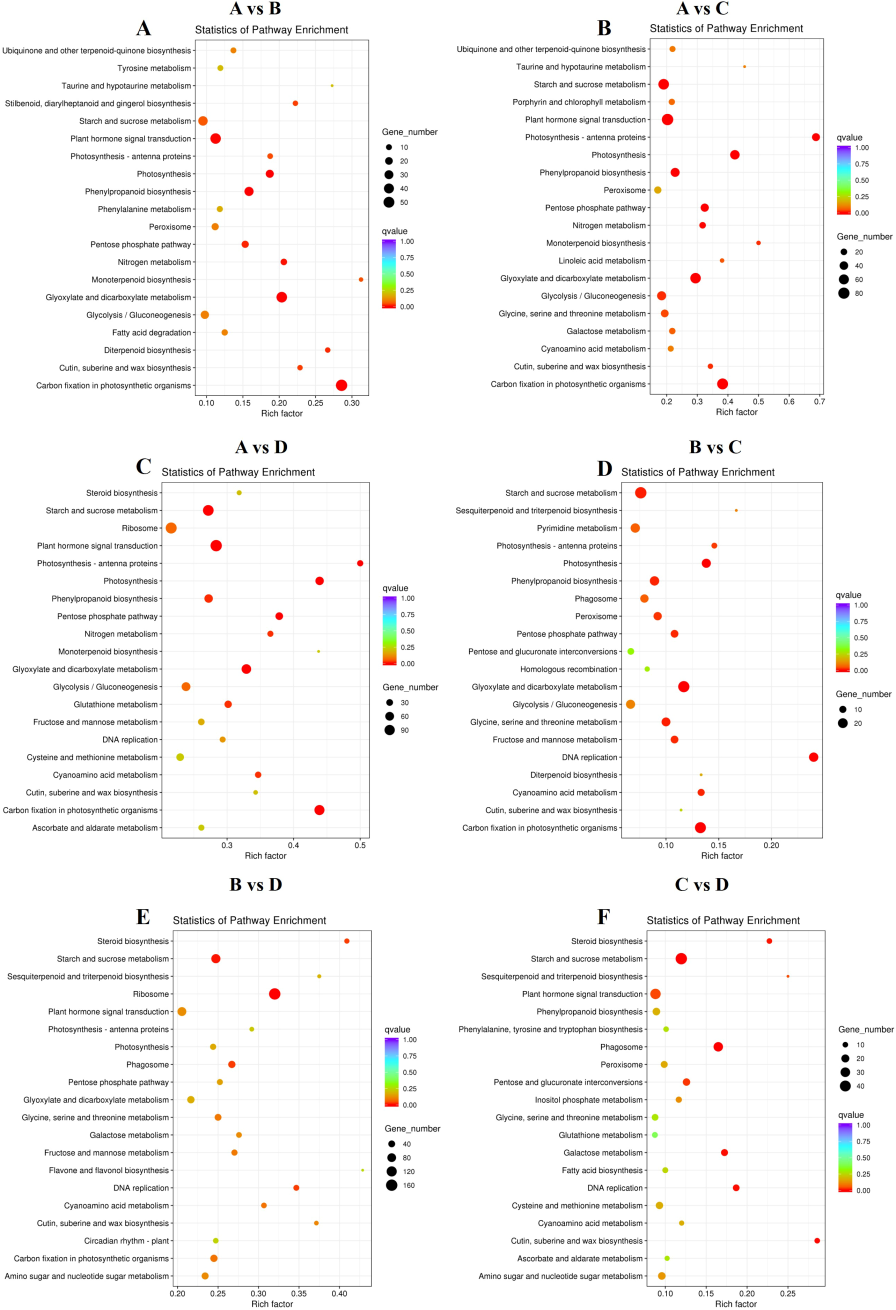
Top 20 significantly enriched KEGG pathways. **(A)** “A vs. B” **(B)** “A vs. C” **(C)** “A vs. D” **(D)** “B vs. C” **(E)** “B vs. D” **(F)** “C vs. D”. Four developmental stages of kiwifruit leaf. **(A)** bud; **(B)** bronze; **(C)** pale-green; **(D)** mature.

To identify the key DEGs involved in the four leaf development stages, six pairwise comparisons (A-B, A-C, A-D, B-C, B-D, and C-D) were conducted. Subsequent sample groups in all comparison combinations served as the internal reference groups. A total of 14,595 genes were identified among all samples using the criteria |log_2_fold-change| ≥ 1 and FDR < 0.01 (**Supplementary Table 4**). Among these comparison groups, the greatest number of DEGs (9,677) was found between the D and A libraries, in which 5,544 and 4,133 unigenes were upregulated and downregulated, respectively. Conversely, the comparison of the C and B libraries revealed the lowest number of DEGs (1839), with 1,044 and 795 unigenes upregulated and downregulated, respectively (**Figure 4B**). Furthermore, 97 DEGs, 29 upregulated DEGs, and 29 downregulated DEGsoverlapped among the three sets of comparisons (A-B, B-C, and C-D) (**Figure 4C**), suggesting that these common genes exert important effects in terms of facilitating the transition of growing leaves (sinks) to mature ones (sources) through transcriptional regulation. Alterations in gene expression at each leaf development stage are illustrated in the heatmap (**Figure 4D**). Stages A and D were significantly different. The genes in Cluster 2 presented relatively high expression in mature leaves, whereas those in Cluster 3 presented relatively high expression in growing leaves, with opposite gene expression profiles during leaf development. To further explore the main functional categories of the DEGs involved in kiwifruit leaf development, the genes whose expression either decreased or increased during leaf development were subjected to GO enrichment analyses. As revealed by the comparison A-B (**Supplementary Table 5**), the GO terms ‘oxidation-reduction process’ (GO:0055114), ‘transcription factor complex’ (GO:0005667), and ‘oxidoreductase activity’ (GO:0016491) were significantly enriched in the BP, CC, and MF categories, respectively. On the basis of the GO annotations of groups B and C (**Supplementary Table 6**), the GO terms ‘microtubule-based movement’ (GO:0007018), ‘microtubule’ (GO:0005874), and ‘microtubule motor activity’ (GO:0003777) were strongly enriched in the BP, CC, and MF categories, respectively. Groups C and D were compared (**Supplementary Table 7**), and the GO terms ‘protein phosphorylation’ (GO:0006468), ‘tubulin complex’ (GO:0045298), and ‘protein kinase activity’ (GO:0004672) were closely related to the BP, CC, and MF categories, respectively. Furthermore, the screened DEGs were analyzed using KEGG pathway enrichment. The significantly enriched KEGG pathway term ‘starch and sucrose metabolism’ was present in all six comparisons (**Figures 5A–F**). The ‘plant hormone signal transduction’ pathway was associated with the A-B, A-C, A-D, B-D, and C-D comparison groups but not with the B-C group comparison (**Figures 5A–F**). In addition, the top 5 KEGG pathways (p < 0.05) corresponded to the upregulated and downregulated DEGs in the three comparisons (A vs. B, A vs. C, A vs. D) (**Table 3**). The downregulated genes were significantly enriched in ‘photosynthesis’, ‘glyoxylate and dicarboxylate metabolism’, and ‘carbon fixation in photosynthetic organisms’ when B, C, and D were compared with A, whereas the upregulated genes were closely related to ‘starch and sucrose metabolism’ and ‘plant hormone signal transduction’. Consequently, the above pathways were inferred to be crucial for leaf development, which was consistent with the results of the GO analysis.

**Table 3:** Top 5 KEGG pathways in which the DEGs were enriched (p < 0.05).

Combination name	KEGG pathway	Down_ number	Up_ number	DEG_ number	Total_ number	P value
A vs B	Carbon fixation in photosynthetic organisms	44	12	56	196	2.18E-19
	Glyoxylate and dicarboxylate metabolism	46	1	47	231	8.23E-12
	Plant hormone signal transduction	7	39	46	409	7.48E-05
	Starch and sucrose metabolism	15	20	35	368	7.48E-05
	Phenylpropanoid biosynthesis	4	28	32	202	2.59E-06
A vs C	Plant hormone signal transduction	14	69	83	409	9.10E-07
	Carbon fixation in photosynthetic organisms	60	15	75	196	6.28E-17
	Starch and sucrose metabolism	39	31	70	368	4.09E-05
	Glyoxylate and dicarboxylate metabolism	66	2	68	231	4.24E-11
	Photosynthesis	50	2	52	123	1.81E-13
A vs D	Plant hormone signal transduction	17	99	116	409	5.04E-07
	Ribosome	14	94	108	500	0.007674
	Starch and sucrose metabolism	43	57	100	368	1.38E-05
	Carbon fixation in photosynthetic organisms	70	16	86	196	1.25E-12
	Glyoxylate and dicarboxylate metabolism	67	9	76	231	5.63E-07

### 3.5 Identification of the key DEGs responsible for sugar metabolism

Sugars produce the main primary metabolites with various bioactivities within kiwifruit leaves, which have important effects on the growth and development of leaves and fruits. Therefore, the relationships of the DEGs enriched in starch and sucrose biosynthesis using the KEGG database analysis were investigated in the significantly enriched pathways. The A-B, A-C, and A-D groups included 35 (20 upregulated and 15 downregulated), 70 (31 upregulated and 39 downregulated), and 100 (57 upregulated and 43 downregulated) starch and sucrose metabolism genes, respectively (**Table 3**). Interestingly, as the leaf development stage progressed, markedly more DEGs were enriched in each pathway, indicating that changes in gene levels led to markedly altered starch and sucrose levels and percentages.

**Figure 6:**
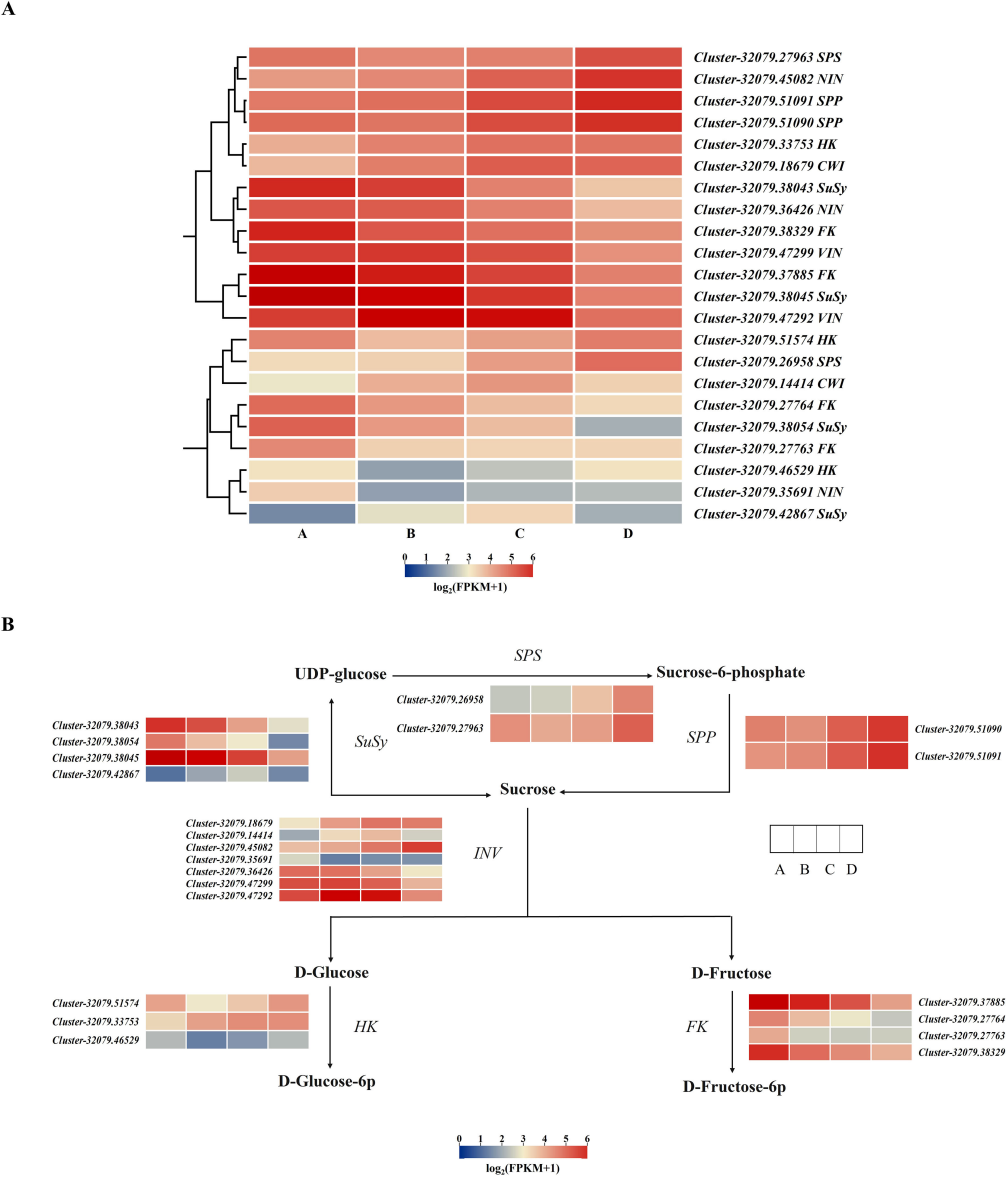
Heatmap showing the DEGs associated with sucrose metabolism-related enzymes. **(A)** The heatmap obtained using TBtools according to log_2_FPKM+1. The right column presents the relevant gene IDs and names. The color bar represents the gene level. **(B)** The simplified sucrose biosynthesis and inversion pathway. The columns and rows in the heatmap represent the samples and genes, respectively. SPS, sucrose phosphate synthase; SuSy, sucrose synthase; SPP, sucrose phosphatase; INV, invertase; HK, hexokinase; FK, fructokinase. Four developmental stages of kiwifruit leaf. **(A)** bud; **(B)** bronze; **(C)** pale-green; **(D)** mature.

**Figure 7:**
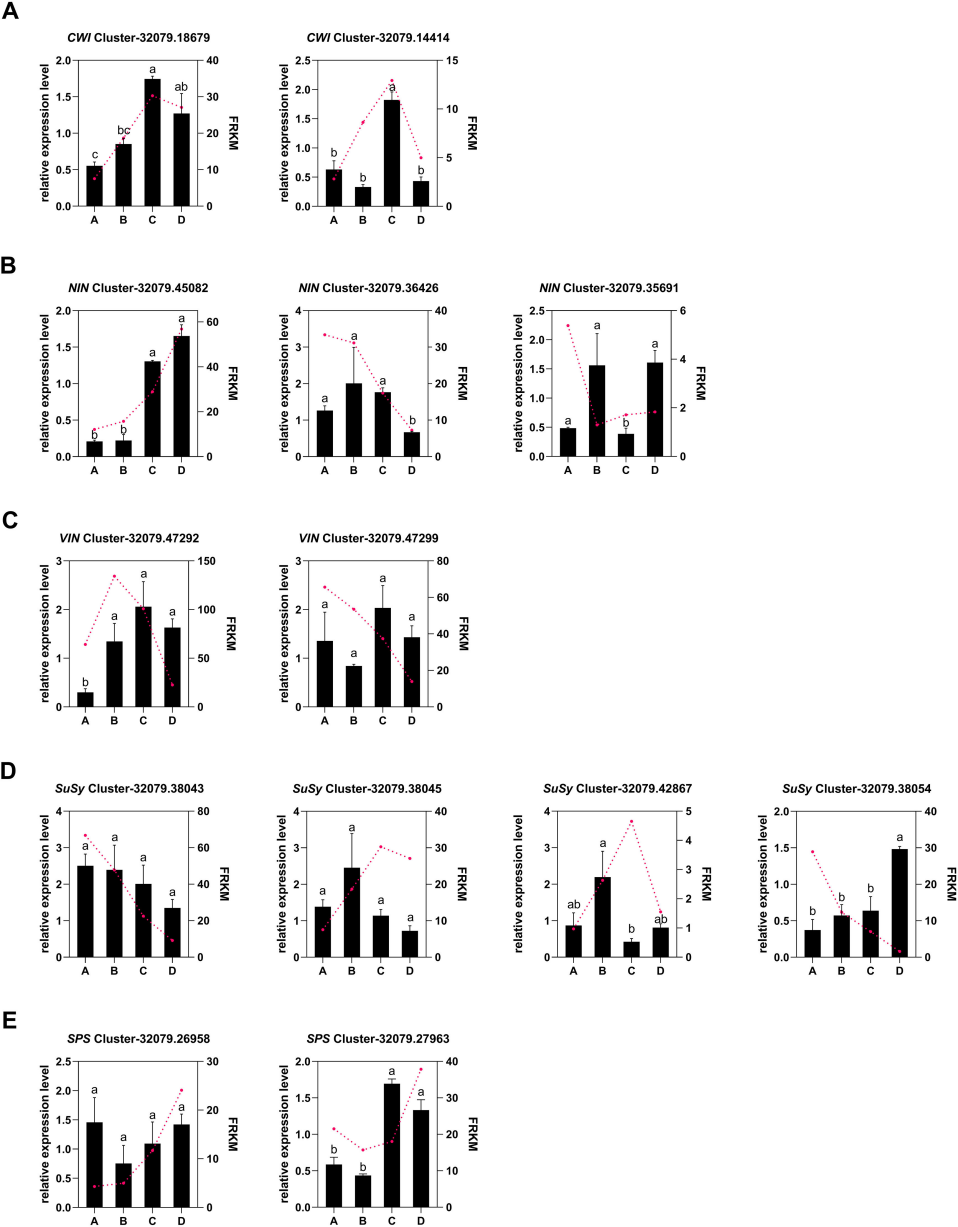
Changes in the expressions of sucrose-metabolizing genes from stages A to D during the kiwifruit leaf development process, as determined through qPCR and RNA-seq. **(A)** two cell wall invertase genes; **(B)** three alkaline/neutral invertase genes; **(C)** three vacuolar invertase genes; **(D)** four sucrose synthase genes; **(E)** two sucrose phosphate synthase genes. The data presented are the means ± SEs from three replicates. The different letters on the top of the bars indicate significant differences (P < 0.05) across the different leaf development stages. Four developmental stages of kiwifruit leaf. **(A)** bud; **(B)** bronze; **(C)** pale-green; **(D)** mature.

A total of 22 DEGs associated with metabolism-related enzymes (VIN, NIN, CWI, SPS, SPP, FK, HK, and SuSy) were retrieved from the whole DEG pool (**Supplementary Table 8**). A cluster heatmap analysis classified these 22 DEGs into two groups on the basis of their corresponding expression: class I contained 13 DEGs that were highly expressed in leaves, whereas class II had 9 DEGs that were weakly expressed in the leaves. Among the 13 highly expressed DEGs, those encoding SPP, SPS, CWI, and HK were expressed during the leaf development process, particularly in stage D. The expression levels of two SuSy DEGs, two VIN DEGs, and two FK DEGs decreased from A to D (**Figure 6A**). SuSy plays an important role in sucrose metabolism and is responsible for catalyzing sucrose production and decomposition. According to the results of this study, all four *SuSy* genes in kiwifruit leaves decreased markedly from stages C to D, and *Cluster-32079.38043* and *Cluster-32079.38045* were highly expressed in the early leaf development stages (A to B), as depicted in **Figure 6B** and **Figure 7D**. Furthermore, two SPP genes (*Cluster-32079.51090* and *Cluster-32079.51091*) and two SPS genes (*Cluster-32079.26958* and *Cluster-32079.27963*) were identified to be involved in sucrose synthesis, and all of these genes were upregulated during leaf development (**Figures 6B**, **7F**). The three invertase genes presented different expression patterns across the four leaf developmental stages. Both *AcCWI* genes (*Cluster-32079.18679* and *Cluster-32079.14414*) presented high expression in stage C and low expression in stage D of *Cluster-32079.14414*, conforming to the variation trend of CWI activity (**Figures 6B**, **7A**). The expressions of three *AcNIN* genes changed during stages A-D, and *Cluster-32079.45082* presented persistently elevated expression, whereas the expressions of *Cluster-32079.36426* and *Cluster-32079.35691* fluctuated to varying degrees (**Figures 6B**, **7B**). The expressions of both *AcVIN* genes (*Cluster-32079.47292* and *Cluster-32079.47299*) decreased from B to D, but that of *Cluster-32079.47292* was considerably low at stage A (**Figures 6B**, **7C**). Furthermore, four *FK* genes and three *HK* genes were also identified, and four of these genes were downregulated, whereas the majority of the *HK* genes (*Cluster-32079.51574* and *Cluster-32079.33753*) were upregulated during leaf development (**Figure 6B**).

A qRT-PCR analysis was conducted to validate the gene expression profiles derived from RNA-Seq data, revealing 13 DEGs related to starch and sucrose biosynthesis. These included five key genes linked to sucrose metabolism:*AcVIN*, *AcNIN*, *AcCWI*, *AcSPS*, and *AcSuSy* (**Supplementary Table 9**). Overall, the sucrose metabolism gene expression patterns obtained from this RT-qPCR analysis were consistent with those obtained from the RNA-seq analysis, except for small differences in expression levels at certain time points (**Figures 7A–E**). Therefore, these RNA-seq data were regarded as dependable. Correlation analysis indicated that among the 13 genes associated with sucrose metabolism, only four genes, viz. *AcNIN* (*Cluster-32079.45082*), *AcVIN* (*Cluster-32079.47292*), and *AcSuSy* (*Cluster-32079.38045* and *Cluster-32079.42867)* were strongly associated with the corresponding enzyme activities (**Table 4**).

**Table 4:** Correlation coefficients for the associations between enzyme activity and gene expression during the development of kiwifruit leaves.

Gene expression	Enzyme activity	Correlation coefficient
*Cluster-32079.18679*	CWI	0.94748
*Cluster-32079.14414*		0.920487
*Cluster-32079.45082*	NIN	0.956566^a^
*Cluster-32079.36426*		-0.680791
*Cluster-32079.35691*		0.365952
*Cluster-32079.47292*	VIN	0.999195^b^
*Cluster-32079.47299*		0.458223
*Cluster-32079.26958*	SPS	0.246985
*Cluster-32079.27963*		0.590533
*Cluster-32079.38043*	SSS	-0.695188
*Cluster-32079.38045*		-0.589986
*Cluster-32079.42867*		-0.59873
*Cluster-32079.38054*		0.504482
*Cluster-32079.38043*	SSC	0.563464
*Cluster-32079.38045*		0.950327^a^
*Cluster-32079.42867*		0.985461^a^
*Cluster-32079.38054*		-0.372576

^a^P < 0.05; ^b^P < 0.01. SPSS Statistics 22 was used for correlation analysis.

## 4 Discussion

Sucrose is the photosynthetic end product in most fruit tree species and the major carbohydrate form delivered from source (leaf) to sink tissue, thereby offering the energy and material foundation for organ growth and development in plants (Koch, 1984). Left development involves sink status (bud-young leaf) transitioning into source status (mature leaf), which is accompanied by distinct changes in morphological traits, photosynthetic capacity, sugar content, enzyme activities, and gene expression patterns (Daudet et al., 2002; Knoblauch and Peters, 2017; Ribeiro et al., 2021). In this study, stages A-C represented immature leaves, whereas stage D represented mature leaves. It has been previously reported that increased hexose contents are advantageous for leaf development, as they play a crucial role in promoting rapid cell expansion and division (da Silva Bonome et al., 2011). Elevated hexose contents also support the regulation of cell wall polymer synthesis, which is essential for cell wall elongation and facilitates cell development (Cosgrove, 2005). In this study, the contents of hexoses (glucose and fructose) in kiwifruit leaves increased during leaf development and were greater than those of sucrose at all stages (**Figure 2**). These results were similar to observations reported for immature grapevine leaves and citrus leaves, in which the hexose content represented the majority of the total carbohydrates (Patakas, 2000). These findings collectively indicate that elevated levels of hexoses in developing leaves may facilitate water uptake, enabling the establishment of high cell turgor pressure that facilitates rapid leaf expansion. Additionally, the content of glucose, a photosynthesis-derived direct product, increased in stages C and D relative to its levels in stages A and B (**Figure 2**). These results align with those reported previously for citrus leaves, supporting the notion that when a plant leaf is halfway through its growth, it achieves a positive carbon balance and becomes self-sufficient (Turgeon, 2006). Furthermore, the leaf sucrose and starch contents in stage D increased markedly in stages A-C (**Figure 2**). In most plants, sucrose is the main photo-assimilate transported to sink organs from source tissues, and starch is a temporary photo-assimilate reserve in the leaves of fruit crops, including kiwifruit (Koch, 1984). The leaves in stage D accounted for the completely functional sink source, whereas those in stage C were independent and in a transition stage; they did not contribute greatly as sinks. Several studies have shown that, in most plant species, the starch content in leaves is greater than that of sucrose, such as in *Flaveria trinervia* (Spreng.) C. Mohr (Lunn and Hatch, 1995), maize (Huber, 1989), cucumber (Huber, 1989), and young common oak (Alaoui-Sossé et al., 1996). However, studies by Zhu et al (Zhu et al., 2018), Zhang et al. (2015), and Ould-Ahmed et al. (2017) have demonstrated that in some plants, the sucrose content is similar to or greater than the starch content during certain stages of leaf development. According to the findings of this study, the starch-sucrose ratio was about 4.0 in mature kiwifruit leaves, indicating that kiwifruit typically accumulate high concentrations of starch.

Changes in leaf sugar composition and levels can be determined based on the changes in the activities of enzymes related to sucrose metabolism, as enzyme activity is a key function necessary for plant growth. This study revealed that four sucrose-cleaving enzymes, namely, VIN, NIN, CWI, and SuSy, had high activities in immature leaves in SSC, which is consistent with the increased hexose contents noted in growing leaves (**Figures 3A–D**). Similar results have been reported for peach (Merlo and Passera, 1991) and *Hevea* leaves (Zhu et al., 2018), with VIN, NIN, and SSC activities, as well as the hexose level, which peaks in rapidly expanding leaves. The literature has not consistently demonstrated that VIN is the main enzyme involved in sucrose catabolism in growing or mature leaves, whereas NIN and CWI activities have been reported to dramatically decrease or are often reported as undetected (Singh and Luthra, 1988; Huber, 1989; Alaoui-Sossé et al., 1996; Winter and Huber, 2000). In this study, however, SuSy was the major enzyme related to sucrose breakdown during the early stages (A and B) of leaf development, and NIN and CWI exhibited activities comparable to those of VIN during the later stages (C and D) of leaf development (**Figures 3A–C**), suggesting that these four enzymes play important roles in different kiwifruit leaf development stages. Additionally, sucrose levels were previously reported to be positively related to SSS and SPS activities in certain species (Burger and Schaffer, 2007). The results for kiwifruit leaves, similar to those reported for *Hevea* leaves (Zhu et al., 2018), revealed that SSS activity was greater than that of SPS at different leaf development stages, and the former was also involved in sucrose accumulation, indicating that SuSy, not SPS, was the rate-limiting enzyme for sucrose synthesis.

Plant sugars can be used as metabolites or signal molecules to initiate signaling pathways, leading to gene expression changes and physiological adaptations (Wind et al., 2010). In order to further understand the molecular foundation underlying the sugar level differences in the developing leaves of kiwifruit, transcriptome sequencing was carried out to identify the key genes regulating sugar levels and enzyme activities during leaf development in kiwifruit. The DEGs in the AvsB, AvsC, and AvsD groups were markedly associated with starch and sucrose metabolism (**Table 3**); moreover, 22 sucrose metabolism-related genes INV, SuSy, SPS, SPP, HK, and FK were identified in the KEGG analysis (**Figures 6A, B**). SPS, SPP, and SuSy were involved in sucrose resynthesis. Two SPP genes (*Cluster-32079.51090* and *Cluster-32079.51091*) and two SPS genes (*Cluster-32079.26958* and *Cluster-32079.27963*) were upregulated during leaf development (**Figures 6B**, **7E**). Interestingly, the activity of SPS was correlated to the variations in its mRNA levels throughout the leaf development process, suggesting that SPS contributes to sucrose synthesis in the leaves. However, the expression levels of the four *AcSuSy*s genes analyzed were not significantly related to SSS enzyme activity (**Table 4**). Similar observations have been reported for sugar beet (Pavlinova et al., 2002), indicating that post-translational enzyme modification is important for regulating SuSy activity. SuSy can reversibly hydrolyze sucrose, whereas INV (CWIN, NI, and SAI) enzymes are responsible for irreversible sucrose hydrolysis. Among the 13 genes, the expression levels of *AcCWI* (*Cluster-32079.18679*), *AcNIN* (*Cluster-32079.45082*), and *AcVIN* (*Cluster-32079.47292*) in stages A to D were increased (**Figures 6B**, **7A–C**), whereas those of the two *AcSuSy* genes (*Cluster-32079.38045* and *Cluster-32079.42867*) were decreased (**Figures 6B**, **7D**), which indicated a probable function of these genes in sucrose decomposition. Furthermore, the expression levels of *Cluster-32079.45082*, *Cluster-32079.47292*, *Cluster-32079.38045*, and *Cluster-32079.42867* were significantly correlated to sucrose-hydrolyzing enzyme activity (**Table 3**). These findings were similar to those reported for *Hevea* leaves, in which sucrose synthase and invertase (in the cleavage direction) reportedly exert important effects on kiwifruit (Zhu et al., 2018). Additionally, the expression of the four *FK* genes was downregulated in stages A to B, which was indicative of reduced F6P flux from fructose phosphorylation. However, *HK* genes (*Cluster-32079.51574* and *Cluster-32079.33753*) were upregulated during leaf development (**Figure 6B**). This finding was consistent with the previous reports showing high transcript levels of *HK* genes through glucose signaling (Karve et al., 2008; Sheen, 2014).

In summary, this study is the first to determine the changes in sucrose-metabolizing enzyme activities and gene levels during leaf development, along with sugar content profiles, in kiwifruit leaves at different stages of development. Unlike those in most species, several genes and enzymes involved in sucrose metabolism in kiwifruit leaves present several unique characteristics. SuSy was the primary enzyme for sucrose breakdown during early leaf development (stages A and B), whereas NIN and CWI exhibited activities that were comparable to those of VIN in the later stages (stages C and D). Additionally, SPS was the dominant enzyme involved in sucrose synthesis in most plant leaves. However, in kiwifruit leaves, SuSy, which functions in the synthetic direction, was more active than SPS across all leaf development stages. The comprehensive sugar, enzyme, and transcriptome data reported in the present work constitute a useful resource for identifying sucrose metabolism-related genes and enzymes during the development of kiwifruit leaves.

## Data Availability

The original contributions presented in the study are included in the article/**Supplementary Material**. RNA-seq data were submitted to https://bigd.big.ac.cn/gsub/, accession number CRA025148. Further inquiries can be directed to the corresponding author.
